# Chemical Exposure-Induced Changes in the Expression of Neurotrophins and Their Receptors in the Main Olfactory System of Mice Lacking TRPM5-Expressing Microvillous Cells

**DOI:** 10.3390/ijms19102939

**Published:** 2018-09-27

**Authors:** Abdullah AlMatrouk, Kayla Lemons, Tatsuya Ogura, Wangmei Luo, Chantel Wilson, Weihong Lin

**Affiliations:** Department of Biological Sciences, University of Maryland, Baltimore County, Baltimore, MD 21250, USA; ow06215@umbc.edu (A.A.); kalem1@umbc.edu (K.L.); tatsuya@umbc.edu (T.O.); wangmei.luo@yahoo.com (W.L.); wilson14@umbc.edu (C.W.)

**Keywords:** olfactory epithelial maintenance, real-time qPCR, olfactory sensory neurons, microvillous cells, *Skn-1a/Pou2f3*

## Abstract

Functional maintenance of the mammalian main olfactory epithelium (MOE) is challenging because of its direct exposure to a wide spectrum of environmental chemicals. We previously reported that transient receptor potential channel M5-expressing microvillous cells (TRPM5-MCs) in the MOE play an important role in olfactory maintenance. To investigate the underpinning mechanisms, we exposed transcription factor *Skn-1a* knockout (*Skn-1a^−/−^*) mice lacking TRPM5-MCs, and TRPM5-GFP mice to either vehicle (water) or a mixture of odorous chemicals and chitin for two weeks and analyzed the expression of olfactory signaling proteins using immunolabeling and neurotrophin (NT) and NT receptor (NTR) gene transcripts using real-time quantitative PCR. The chemical exposure did not significantly attenuate the immunolabeling of olfactory signaling proteins. Vehicle-exposed *Skn-1a^−/−^* and TRPM5-GFP mice expressed similar levels of NT and NTR gene transcripts in the MOE and olfactory bulb. Chemical exposure significantly increased MOE expression of *p75NTR* in *Skn-1a^−/−^* mice, while *p75NTR* expression was reduced in TRPM5-GFP mice, as compared to vehicle-exposed mice. Additionally, our RNA in situ hybridization analysis and immunolabeling confirmed MOE expression of most NTs and NTRs. Together, these results indicate that TRPM5-MCs and chemical exposure influence expression of some NTs and NTRs in the MOE and olfactory bulb (OB).

## 1. Introduction

The sense of smell, initiated by the main olfactory epithelium (MOE) in the nasal cavity, provides animals and humans with information vital for their survival, as it enables them to assess food quality, detect pathogens, avoid predators, and find mating partners. Due to its direct contact with the external environment, the MOE is exposed to a wide variety of inhaled chemicals including not only odorants, but also irritants, toxicants, and pathogenic microorganisms. Some of these substances can cause structural and functional damage depending on the type of molecules, exposure levels, and durations [1,2,3]. Chronic exposure to these harmful substances contributes to the development of neurodegenerative diseases such as Alzheimer’s and Parkinson’s diseases [4]. Therefore, the lifelong maintenance of MOE integrity and plasticity for adapting to external changes is essential for animals and humans. However, protective mechanisms against environmental insults are poorly understood, especially under chronic mild exposure, which is known to disturb brain function and behavior [5].

The MOE is primarily made up of olfactory sensory neurons (OSNs), supporting cells (SCs; also referred to as sustentacular cells), basal progenitor cells, and microvillous cells (MCs). These cells differ substantially in terms of morphology and function. MCs can be subdivided based on their morphological features, specific cell marker expression, and functions. Importantly, recent findings have shown that MCs are involved in MOE maintenance. One population of MCs expresses transient receptor potential channel C6 (TRPC6-MCs) and can release neuropeptide Y (NPY) to promote adult neurogenesis [6]. Another population of MCs, which we and other investigators previously characterized, expresses transient receptor potential channel M5 (TRPM5-MCs) [7,8]. Our subsequent study revealed that TRPM5-MCs are chemoresponsive and capable of synthesizing and releasing acetylcholine (ACh), which can modulate the activities of OSNs and supporting cells [9,10]. More recently, we identified an important role for TRPM5-MCs in maintaining olfactory function using mice deficient in the POU homeobox transcription factor gene *Skn-1a* (also known as *Pou2f3*; *Skn-1a^−/−^* mice), in which TRPM5-MCs in the MOE failed to differentiate [11,12]. Under regular housing conditions, *Skn-1a^−/−^* mice did not show significant deficits in odor responses and olfactory-guided behaviors. However, after being housed in an environment with an added odiferous chemical mixture and brief daily exposure to chitin for two weeks, *Skn-1a^−/−^* mice showed a significant reduction in their odor- and pheromone-evoked electro-olfactogram responses. Subsequently, the olfactory ability of guiding mice towards food and the preference reactions towards socially and sexually relevant odorants were significantly compromised. In contrast, control TRPM5- green fluorescent protein (GFP) mice, which have the same C57BL/6 genetic background and were treated under the same conditions did not show any such significant deficits [12]. These results provide evidence that TRPM5-MCs are important for the ability of the MOE to undergo adaptive changes in order to maintain olfactory functions.

Mechanisms underlying the protective functions of TRPM5-MCs have not been explored in detail. Because TRPM5-MCs are not directly involved in olfactory signaling and information processing, it is expected that TRPM5-MCs modulate MOE network activity. In addition to the cholinergic modulation of OSNs and SCs [9,10], TRPM5-MCs may engage other mechanisms that play important roles in MOE maintenance. One such mechanism is adult neurogenesis, which involves globose and horizontal basal cells residing in the basal compartment of the olfactory epithelium [13]. These progenitor cells give rise to immature and mature OSNs as well as non-neuronal SCs and MCs, including TRPC6-MCs and TRPM5-MCs [14,15,16]. Thus, the basal cells play an important role in injury-triggered MOE repair and also adult regeneration for homeostatic maintenance. Since we did not observe major changes in the MOE thickness and olfactory marker protein expression in most MOE regions of *Skn-1a^−/−^* mice examined after the two-week chemical exposure in our previous study [12], the critical involvement of basal cells in chemical exposure-induced functional deficits in *Skn-1a^−/−^* mice is not known.

Another mechanism of MOE maintenance involves neurotrophic factors and their receptors. Neurotrophins (NTs) are a family of polypeptide growth factors important for regulating neuronal survival, proliferation, differentiation, plasticity, and functions in the nervous system, including MOE and olfactory bulb (OB) [17,18,19,20,21,22]. In mammals, members of the four NT families, i.e., the nerve growth factor (NGF), brain-derived neurotrophic factor (BDNF), neurotrophin-3 (NT-3), and neurotrophin-4 (NT-4) families, have been well characterized [23]. Each of the four NTs has a high-affinity preference for the tropomyosin receptor kinase (Trk) subfamily of tyrosine kinase receptors, which include TrkA, TrkB, and TrkC [24]. NGF specifically binds to TrkA. BDNF, and NT-4 bind to TrkB, and NT-3 binds to TrkC and other Trk receptors with low affinity [25]. Additionally, all four NTs bind to the pan-neurotrophin receptor, p75NTR, with similar affinity [26]. By binding to their receptors, NTs activate different downstream pathways, including the extracellular signal-regulated protein kinase (ERK) pathway, phosphatidylinositol-4,5-bisphosphate 3-kinase (PI3K)/protein kinase B(Akt) pathway, and the phospholipase Cγ (PLCγ) pathway [27]. Previous studies have shown the expression of PLCγ1 and γ2 in OSNs [28,29]. In addition, ERK and PI3K, which function as downstream effectors of NT receptors, are functionally expressed in the MOE [30,31].

The expression of NTs and NT receptors (NTRs) in the MOE has been investigated using reverse transcription polymerase chain reaction (RT-PCR), real-time quantitative PCR (qPCR), RNA in situ hybridization (RISH), and immunohistochemical staining [20,21,32,33,34]. However, inconsistent results were observed for some NTs and NTRs, especially BDNF and p75NTR in terms of their protein expression levels and locations (for details, see Feron et al., 2008 [21]). Buckland and Cunningham [34] showed that BDNF was expressed in HBCs, whereas Takami et al. [33] and Feron et al. [21] showed that BDNF was expressed in mature OSNs. The expression of NTs is affected by a myriad of factors, including neurotransmitters [35]. Furthermore, exposure to cigarette smoke has also been shown to influence NT expression, as administering cigarette smoke solution to chemically injured MOE tissue suppressed insulin-like growth factor-1 expression [36].

We sought to investigate the mechanisms underpinning TRPM5-MCs-mediated olfactory functional maintenance. Because chemical exposed *Skn-1a^−/−^* mice exhibited significant reduction in odor-evoked responses without global significant MOE morphological changes [12], we examined the expression of olfactory signaling proteins using immunolabeling. Primarily, we focused on whether the lack of TRPM5-MCs resulted in altered expression of NTs and NTRs directly in the MOE or indirectly in the olfactory bulb (OB) after two-week chemical exposure using real-time qPCR analysis. To be consistent with our published study, we used the same protocol and conditions we previously developed for creating a relatively chronic mild exposure condition. The treatment was intended to challenge (but not overwhelm) the MOE’s ability to maintain its integrity in control mice where TRPM5-MCs are present and functioning in the MOE, so that we could assess the effects of the exposure on *Skn-1a^−/−^* mice lacking TRPM5-MCs. With this treatment, we discovered that the adaptive plasticity of the MOE was compromised in *Skn-1a^−/−^* mice but not in the control group of TRPM5-GFP mice of the same genetic background, without inducing significant morphological changes in most of the MOE examined in both strains [12]. The odorous chemicals used for the two-week chemical exposure were selected largely on the basis of their widespread use in various industrial applications, the possibility of them being inhaled by animals in natural conditions, and their relevance to occupational health/exposure toxicology [37]. For example, propionic acid was selected because it can be produced by bacteria commonly found in the nasal mucosa [38] and TRPM5-MCs respond to chemicals in bacterial lysate [9]. Triethylamine was chosen due to its widespread use in chemical synthesis. Additionally, we subjected mice to brief daily exposure to chitin via inhalation because chitin is found abundantly in nature as a major component of cell walls in fungi, as well as in the exoskeletons of arthropods and insects. Chitin is also an allergen that can cause inflammation after nasal administration [39]. We also used RNA in situ hybridization (RISH) analysis to confirm the expression of NT and NTR gene transcripts in the MOE and immunolabeling of p75NTR for verification. Together, our results provide evidence that the expression of some NT and NTR gene transcripts is influenced by chemical exposure and TRPM5-MCs in the MOE, providing a potential mechanism underlying the protective function of TRPM5-MCs.

## 2. Results

### 2.1. Immunolabeling of Olfactory Signaling Proteins in Vehicle- and Chemical-Exposed TRPM5-GFP and Skn-1a^−/^^−^ Mice

**Figure 1:**
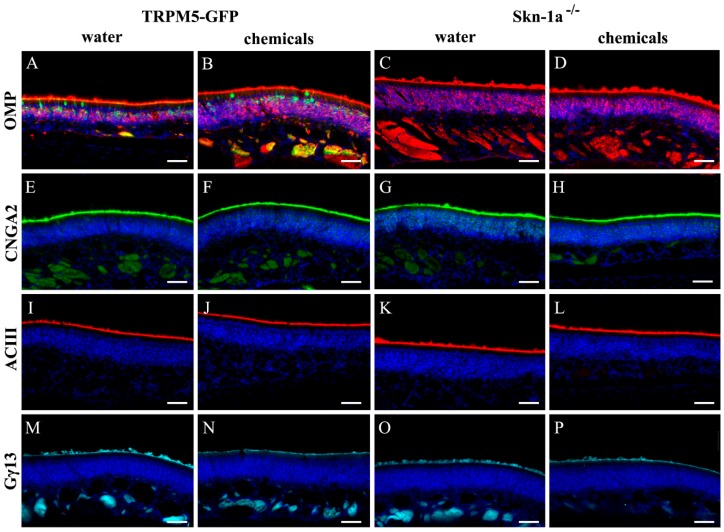
Immunolabeling of olfactory marker protein (OMP) and signaling proteins in TRPM5-GFP and *Skn-1a^−/−^* mice. (**A**–**D**) Immunolabel of OMP (red) in mature OSNs. TRPM5 expression in TRPM5-MCs and TRPM5-expressing OSNs of a TRPM5-GFP mouse are shown in green (GFP+; **A**,**B**). Immunolabels of olfactory signaling protein CNGA2 (green in **E**–**H**) and ACIII (red in **I**–**L**), and G-protein Gγ13 (cyan in **M**–**P**) are shown. (**A**,**E**,**I**,**M**) Vehicle (water)-exposed TRPM5-GFP mice. (**B**,**F**,**J**,**N**) chemical-exposed TRPM5-GFP mice. (**C**,**G**,**K**,**O**) Vehicle (water)-exposed *Skn-1a^−/−^* mice. (**D**,**H**,**L**,**P**) chemical-exposed *Skn-1a^−/−^* mice. For each antibody, images were taken under the same exposure time and from similar septal regions of coronal main olfactory epithelium (MOE) sections processed under the same conditions. DAPI staining is shown in blue. Scale bar: 40 μm.

The canonical olfactory signal transduction is mediated by a G-protein coupled cAMP pathway. To investigate whether chemical exposure altered the protein expression of key elements of the pathway, we immunolabeled MOE sections of vehicle- and chemical-exposed TRPM5-GFP and *Skn-1a^−/−^* mice using antibodies against cell markers and key signaling proteins. We found intense immunoreactivity of OMP in apical cilia layer and mature OSNs (Figure 1A–D). As expected, the antibodies against cyclic nucleotide-gated channel A2 subunit (CNGA2) (Figure 1E–H), adenynylyl cyclase 3 (AC3) (Figure 1I–L), and Gγ13 (Figure 1M–P), which is the dominant G-protein γ subunit [40,41], strongly labeled the olfactory cilia layer, where olfactory signaling transduction takes place. We found that there was no apparent difference between the TRPM5-GFP and *Skn-1a^−/−^* mice. Also, there was no obvious reduction in the intensity of immunolabeling in sections obtained from chemical-exposed *Skn-1a^−/−^* mice. Since the MOE sections examined were selected from the middle to posterior MOE, which accounts for the majority of MOE surface area, our results indicate that the two-week chemical exposure did not alter significantly morphology, the thickness of the mature OSNs or ciliary expression of signal transduction proteins in at least most of the MOE.

### 2.2. Quantitative Analysis of the Expression of NT and NTR Gene Transcripts in the MOE and OB of Vehicle-Exposed Skn-1a^−/−^ and TRPM5-GFP Mice 

**Figure 2:**
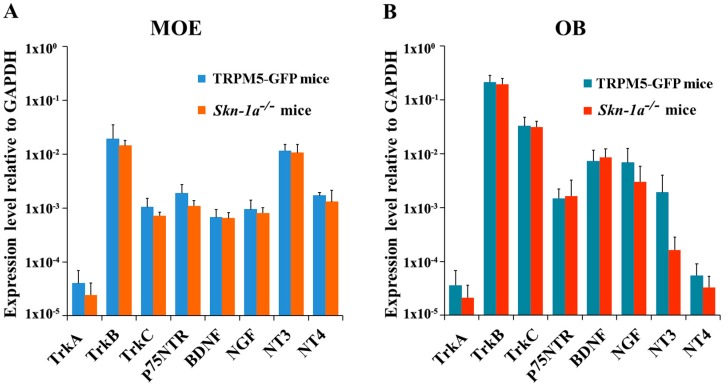
Expression levels of neurotrophin and neurotrophin gene transcripts in the MOE and OB of vehicle-exposed TRPM5-GFP and *Skn-1a^−/−^* mice. Real-time qPCR was performed using total RNA extracted from (**A**) olfactory turbinate tissue consisting primarily of the MOE, and (**B**) the OB. The expression levels are plotted relative to the expression of the *Gapdh* reference gene. (**A**) Expression levels of each NT and NTR in the MOE of TRPM5-GFP and *Skn-1a^−/−^* mice. Similar expression patterns across the NTs and NTRs were found in both TRPM5-GFP and *Skn-1a^−/−^* mice with *NT-3* and *TrkB* expression levels being the highest among NTs and NTRs, respectively. No statistically significant differences were detected in the expression of each gene in the MOE between TRPM5-GFP and *Skn-1a^−/−^* mice (two-tailed independent *t*-test, *p* > 0.05, *n* = 6 mice). (**B**) Expression levels of NTs and NTRs in the OB of TRPM5-GFP and *Skn-1a^−/−^* mice. Among the NTRs examined, *TrkB* showed the highest expression in both TRPM5-GFP and *Skn-1a^−/−^* mice (one-way ANOVA with Tukey’s post-hoc test, *p* < 0.05, *n* = 6). No statistically significant differences were detected in the expression of each gene in the OB between TRPM5-GFP and *Skn-1a^−/−^* mice (two-tailed independent *t*-test, *p* > 0.05, *n* = 6 mice). Bar graphs represent the mean ± standard deviation (SD).

To investigate whether the MOE of *Skn-1a^−/−^* mice expresses NT and NTR gene transcripts at levels similar to those in the TRPM5-GFP mice, we performed qPCR for all of these molecules using mRNA extracted from the MOE tissues of vehicle (water)-exposed mice. The relative expression levels, which were normalized to glyceraldehyde 3-phosphate dehydrogenase (*Gapdh*) expression, are shown in Figure 2A. Although the levels of the NT and NTR gene transcripts varied considerably, both TRPM5-GFP and *Skn-1a^−/−^* mice showed similar expression trends. Among the NTRs, *TrkB* showed the highest expression (one-way analysis of variance [ANOVA] with Tukey’s post-hoc analysis, *p* < 0.001, *TrkB* versus all other receptors, *n* = 6 mice for both control and *Skn-1a^−/−^* mice), which was approximately 100 times more than that of *TrkA*, the receptor with the lowest expression. Among the NTs, expression of *NT-3* gene transcript was approximately 10 times higher than that of *BDNF*, *NGF*, and *NT-4*. Statistical analysis revealed a significantly higher expression level of *NT-3* than that of *BDNF* and *NGF* in the MOE of TRPM5-GFP mice (one-way ANOVA with Tukey’s post-hoc analysis, *NT-3* vs. *BDNF*: *p* = 0.032; *NT-3* vs. *NGF*: *p* = 0.040, *n* = 6). In *Skn-1a^−/−^* mice, *NT-3* expression was significantly higher than that of all other NTs (one-way ANOVA with Tukey’s post-hoc analysis, *NT-3* vs. *BDNF*: *p* < 0.001; *NT-3* vs. *NGF*: *p* < 0.001; *NT-3* vs. *NT-4: p* < 0.001, *n* = 6 mice). These results revealed the relative abundances of NTs and NTRs in the mouse MOE.

By comparing the expression of NTs and NTRs between the TRPM5-GFP and *Skn-1a^−/−^* mice, we found no significant difference between the expression of NTs and NTRs (two-tailed independent *t*-test, *n* = 6 mice). Thus, in the vehicle-exposed groups, the MOE of *Skn-1a^−/−^* mice showed the same expression trends for both NTs and NTRs and similar levels of NTs and NTRs, compared with those in TRPM5-GFP mice. These results provide evidence that the lack of TRPM5-MCs in *Skn-1a^−/−^* mice does not globally impact the expression of these genes in the MOE.

NTs can be transported in a retrograde or anterograde manner between the OB and the MOE [42,43]. Therefore, we quantified the gene-transcript levels of NTs and NTRs in the OB of vehicle-exposed TRPM5-GFP and *Skn-1a^−/−^* mice. Our qPCR results showed the same expression trends across the NTs and NTRs and that TrkB was expressed at significantly higher levels than the other NTRs in both the TRPM5-GFP and *Skn-1a^−/−^* mice (one-way ANOVA with Tukey’s post-hoc analysis, *n* = 6 mice, *p* < 0.001, *TrkB* versus all other receptors; Figure 2B). Furthermore, the expression level of NTs and NTRs was not significantly different than that in the TRPM5-GFP mice. Although the expression of *NT3* in water exposed *Skn-1a^−/−^* tended to be elevated compared to water exposed TRPM5-GFP mice, this difference did not reach statistical significance during analysis. Therefore, the lack of TRPM5-MCs in the MOE did not alter the expression of NTs and NTRs in the OB in vehicle (water)-exposed groups.

### 2.3. Changes in the Expression Levels of NTs and NTRs in the MOE of TRPM5-GFP and Skn-1a^−/−^ Mice after a Two-Week Chemical Exposure

**Figure 3:**
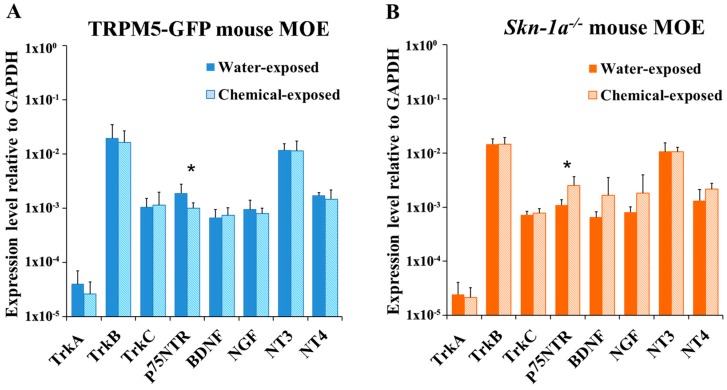
Chemical exposure induced differential alterations in NT and NTR expression in the MOE of TRPM5-GFP and *Skn-1a^−/−^* mice. Real-time qPCR analysis and comparison of the expression of NTs and NTRs in the MOE of mice exposed to either vehicle (water) or a chemical mixture for 2 weeks. *Gapdh* was detected as the reference gene. (**A**) Relative expression levels of NTs and NTRs in TRPM5-GFP mice. No significant differences in the expression levels of NTs or NTRs were observed in the MOE of chemical-exposed mice compared to water-exposed mice except for *p75NTR* expression, which decreased significantly in the MOE of the chemical-exposed group. (**B**) Relative expression levels of NTs and NTRs in *Skn-1a^−/−^* mice. The expression of *p75NTR* in the MOE of the chemical-exposed group was significantly higher than in the vehicle-exposed group. * *p* < 0.05, two-tailed independent *t* test, *n* = 6. Bar graphs represent the mean ± SD.

We performed the same real-time qPCR analysis using RNA extracted from two-week chemical exposed TRPM5-GFP and *Skn-1a^−/−^* mice. We found that the chemical exposure did not significantly change NT or NTR expression in the MOE of TRPM5-GFP mice, except for *p75NTR* expression, which decreased significantly compared to that in the MOE of vehicle-exposed TRPM5-GFP mice (two-tailed independent *t*-test, *p* = 0.034, *n* = 6; Figure 3A). On the other hand, after the chemical exposure, *Skn-1a^−/−^* mice showed significantly increased expression of *p75NTR* (two-tailed independent *t*-test, *p* = 0.018, *n* = 6; Figure 3B). In addition, expression levels of ligands of *p75NTR*, *BDNF* and *NGF*, also increased after the chemical exposure, although this increase was not statistically significant (two-tailed independent *t*-test, *p* > 0.05, *n* = 6; Figure 3B). For a more thorough comparison, we performed two-way ANOVA and analyzed the interaction between mouse line (TRPM5-GFP or *Skn-1a^−/−^*) and exposure (two-week chemical exposure or two-week vehicle exposure) with the expression level of NTs and NTRs. The analysis revealed that the expression of *p75NTR* and NT-4 was significantly affected by the absence of TRPM5-MCs and two-week chemical exposure (two-way ANOVA, *p* = 0.001 and *p* = 0.046, respectively, *n* = 6). Thus, the MOE responded to chemical exposure differently in terms of *p75NTR* and NT-4 expression in *Skn-1a^−/−^* mice lacking TRPM5-MCs, implying that TRPM5-MCs might maintain MOE olfactory function in part via changes in the expression levels of NTs and NTRs.

### 2.4. Regional Changes in the Expression Levels of NTs and NTRs after a Two-Week Chemical Exposure in the Anterior and Posterior MOE of TRPM5-GFP and Skn-1a^−/−^ Mice

**Figure 4:**
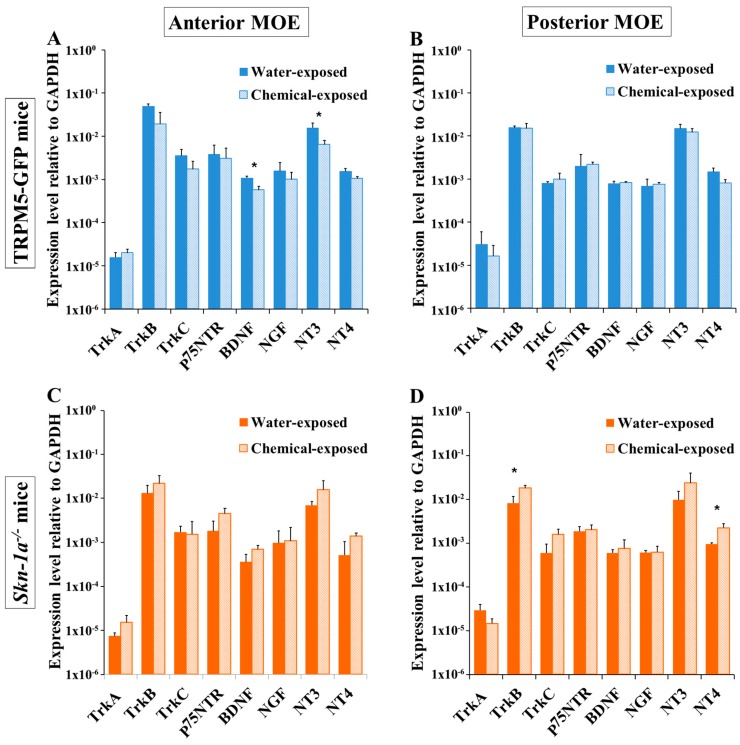
Regional differences in the effects of chemical exposure on MOE NT and NTR gene-expression levels in TRPM5-GFP and *Skn-1a^−/−^* mice. Real-time qPCR was performed to analyze the expression of NTs and NTRs in the anterior and posterior MOEs of mice after a two-week exposure to either vehicle (water) or a chemical mixture. The plots show the NT and NTR mRNA-expression levels relative to that of the reference gene, *Gapdh*. (**A**) NT and NTR expression in the anterior MOE of TRPM5-GFP mice. *BDNF* and *NT-3* expression levels in chemical-exposed TRPM5-GFP mice decreased significantly, compared to those in water-exposed TRPM5-GFP mice. (**B**) NT and NTR expression in the posterior MOE of TRPM5-GFP mice. Two week chemical exposure did not affect the expression of NTs and NTRs. (**C**) NT and NTR expression in the anterior MOE of *Skn-1a^−/−^* mice. No significant changes were detected. (**D**) NT and NTR expression in the posterior MOE of *Skn-1a^−/−^* mice. The expression levels of *TrkB* and *NT-4* in the posterior MOE of chemical-exposed mice were significantly higher than those in water-exposed mice. * *p* < 0.05, two-tailed independent *t* test, *n* = 3. The bar graphs represent the mean ± SD.

Chemical exposure may affect the anterior MOE more severely than the posterior MOE because of uneven airflow rates and chemical distribution within the nasal cavity [44,45]. To determine whether there is a regionally different impact of the two-week chemical exposure on the expression of NTs and NTRs, we performed qPCR using mRNA extracted from the MOE at a site anterior to the olfactory turbinates and the remaining posterior MOE tissues of TRPM5-GFP and *Skn-1a^−/−^* mice, after a two-week chemical or vehicle exposure. In TRPM5-GFP mice, various levels of reduction in the NT and NTR mRNA-expression levels in the anterior MOE were observed in the chemical-exposed group compared with mice in the water-exposed group, except for *TrkA* (Figure 4A), which showed the opposite trend. The *BDNF* and *NT-3* levels were significantly lower (two-tailed independent *t*-test, *p* = 0.006 for *BDNF* and *p* = 0.049 for *NT-3*, *n* = 3). In the posterior MOE, two-week chemical exposure did not change the expression of most NTs and NTRs (*BDNF*, *NGF, NT-3, TrkB, TrkC, p75NTR*), except for *NT-4* and *TrkA*, which showed lower expression after chemical exposure; although these reductions were not significant (two-tailed independent *t*-test, *p* = 0.055 for *NT-4* and *p* = 0.492 for *TrkA*, *n* = 3 mice; Figure 4B). Thus, a regional difference in the effect of chemical exposure on NT and NTR expression was observed in TRPM5-GFP mice.

In *Skn-1a^−/−^* mice, the average expression levels of *TrkA, TrkB, BDNF, p75NTR, NT-3*, and *NT-4* in the anterior MOE were higher in the two-week chemical-exposed group than that in the vehicle (water)-exposed group; however, the differences were not statistically significant (two-tailed independent *t*-test *p* value ranged from *p* = 0.080 for *BDNF* to *p* = 0.336 for *TrkB*, *n* = 3 mice). The expression levels of *TrkC* and *NGF* remained unchanged (Figure 4C). In the posterior MOE, the expression of *TrkB*, *TrkC*, *NT-3*, and *NT-4* increased, but only the increase in *TrkB* and *NT-4* expression was significant (two-tailed independent *t*-test, *p* = 0.021 and 0.019, respectively, *n* = 3 mice; Figure 4D). Together, these results showed that the effect of a two-week chemical exposure on the gene-expression levels of NTs and NTRs in *Skn-1a^−/−^* mice was different from that in TRPM5-GFP mice.

### 2.5. Changes in the Expression of NTs and NTRs in the OB of TRPM5-GFP and Skn-1a^−/−^ Mice after Two-Week Chemical Exposure

**Figure 5:**
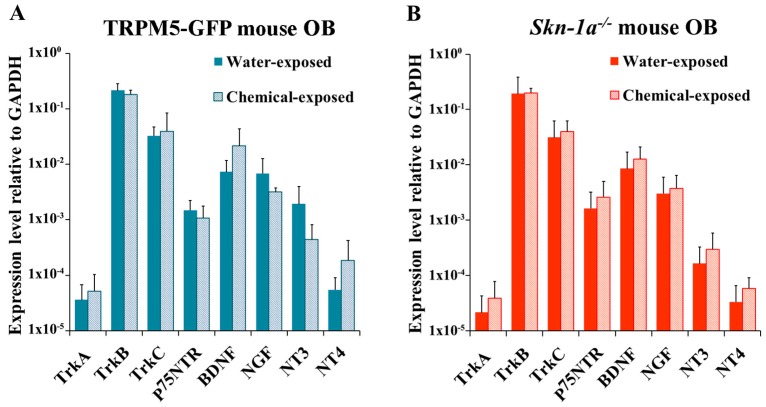
NT and NTR expression levels in the OB of chemical- or vehicle (water)-exposed TRPM5-GFP and *Skn-1*a*^−/−^* mice. The plots show the relative gene expression levels obtained by qPCR analysis using total RNA extracted from the OBs. (**A**) TRPM5-GFP mice exposed to vehicle (water) or chemicals. No significant differences were detected between the two groups (*p* > 0.05, two-tailed independent *t*-test, *n* = 6). (**B**) *Skn-1a^−/−^* mice exposed to vehicle (water) or chemicals. No significant difference was observed between the two groups *(p* > 0.05, two-tailed independent *t*-test, *n* = 6), although the NT and NTR expression levels trended higher in the chemical-exposed group. The bar graphs represent the mean ± SD.

We next tested the effect of two-week chemical exposure on the expression of NTs and NTRs in the OB. In TRPM5-GFP mice, although chemical exposure altered NT and NTR expression, no significant changes were observed (two-tailed independent *t*-test, *p* > 0.05, *n* = 6; Figure 5A). In *Skn-1a^−/−^* mice, mild increases in the expression of all NTs and most NTRs were found, but none of the observed data proved to be statistically significant (two-tailed independent *t*-test, *p* > 0.05, *n* = 6; Figure 5B). We further performed two-way ANOVA to examine the interaction between the mouse line and the two-week chemical exposure with the expression level of NTs and NTRs. This analysis indicated that the effects of the mouse genotype and chemical exposure on NTs and NTRs expression in TRPM5-GFP mice was not different from that in *Skn-1a^−/−^* mice (two-way ANOVA, *p* > 0.05, *n* = 6).

### 2.6. RISH Analysis of the Spatial Distribution of NT and NTR Gene Transcripts in the MOE

**Figure 6:**
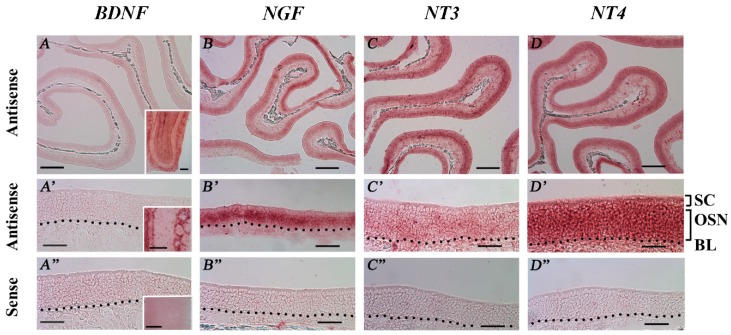
RNA in situ hybridization (RISH) analysis of NT mRNA expression in the MOE of TRPM5-GFP mice. RISH was performed on MOE coronal sections obtained from regularly housed TRPM5-GFP mice with digoxigenin-labeled antisense and sense riboprobes targeting each NT. (**A**–**D**) and (**A’**–**D’**) Low (10×) and high (40×) magnification images, respectively, of the MOE hybridized with the antisense probes for *BDNF, NGF, NT-3*, and *NT-4*. SC: sustentacular/supporting cell layer, OSN: olfactory sensory neuron layer, BL: basal lamina—black dotted line. (**A”**–**D”**) show high-magnification images of sense-probe labeling for *BDNF*, *NGF*, *NT-3*, and *NT-4*, respectively. The sense probes yielded no detectable specific signals in the MOE. Insets in (**A**–**A’’**): RISH images of BDNF mRNA expression in the OB as positive control for the riboprobes used. Scale bars: (**A**–**D**) 200 μm; (**A’**–**D”**) 50 μm.

To confirm the expression of NT and NTR gene transcripts in adult mouse MOE tissues, we performed RISH experiments on coronal nasal sections from regularly housed TRPM5-GFP mice with riboprobes designed specifically for each transcript (Table 1). The antisense riboprobes for *NGF, NT-3*, and *NT-4* yielded positive labeling in the MOE. Low level signals for *NT-3* and *NT-4* were also found in the lamina propria. The sense probes yielded no detectable signals (Figure 6B–D,B’–D’: antisense probe, low and higher magnification, respectively; Figure 6B’’–D’’: sense probes). We tested two antisense riboprobes for *BDNF*; neither produced a positive signal in the MOE, although positive labeling was found in the OB (Figure 6A,A’: antisense probe, low and higher magnification images, respectively; Figure 6A’’: sense probe; insets: OB images). Among the three labeled NTs, a strong RISH signal for *NGF* was found in the OSN layer, whereas the signals for *NT-3* and *NT-4* were diffuse among all the MOE cell layers. These results confirmed the expression of *NGF*, *NT-3*, and *NT-4* gene transcripts in the MOE. Due to the highly similar expression levels of these genes found in our qPCR analyses between the TRPM5-GFP and *Skn-1a^−/−^* mice, we did not perform RISH with the MOE tissues of *Skn-1a^−/−^* mice.

**Figure 7:**
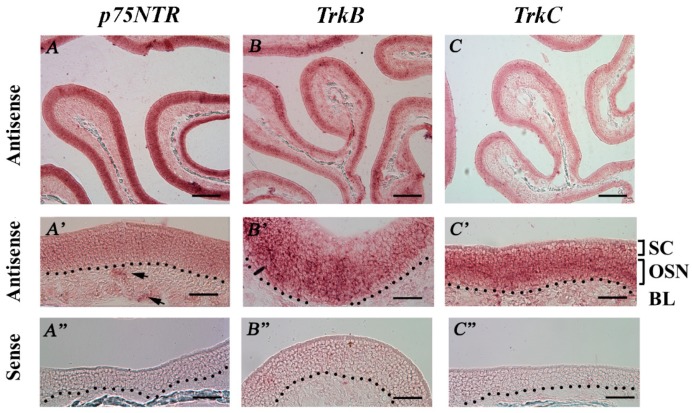
RISH analysis of NTR mRNA expression in the MOE of regularly housed TRPM5-GFP mice. (**A**–**C**) Low-magnification (10×) images showing labels of the antisense probes against *p75NTR*, *TrkB*, and *TrkC*. (**A’**–**C’**) High-magnification (40×) images of antisense probes, correlating with the top row of images. SC: sustentacular/supporting cell layer, OSN: olfactory sensory neuron layer, BL: basal lamina—black dotted line. (**A”**–**C”**) High-magnification (40×) images showing hybridization of the sense probe against *p75NTR, TrkB*, and *TrkC*. Arrows in A’ point to presumed ensheathing cells in the lamina propria. No specific signal was observed for the tested sense probes. Scale bars: (**A**–**C**) 200 μm; (**A’**–**C’’**) 50 μm.

We also performed RISH analysis to study the expression of *p75NTR, TrkB*, and *TrkC* in the MOE of regularly housed TRPM5-GFP mice. We did not test for TrkA because of its low expression in our qPCR experiments. The antisense probes for *p75NTR* and *TrkC* labeled all cell layers in the MOE, including some cells in the lamina propria, whereas labeling with the *TrkB* antisense probe was relatively restricted to the middle MOE layer, where OSN cell bodies reside. The sense probes did not produce specific labels (Figure 7A–C,A’,C’: antisense: low and high magnification, respectively. A’’–C’’: sense probes). These results suggested the expression of three NTRs in the MOE.

### 2.7. Immunolabeling of p75NTR

**Figure 8:**
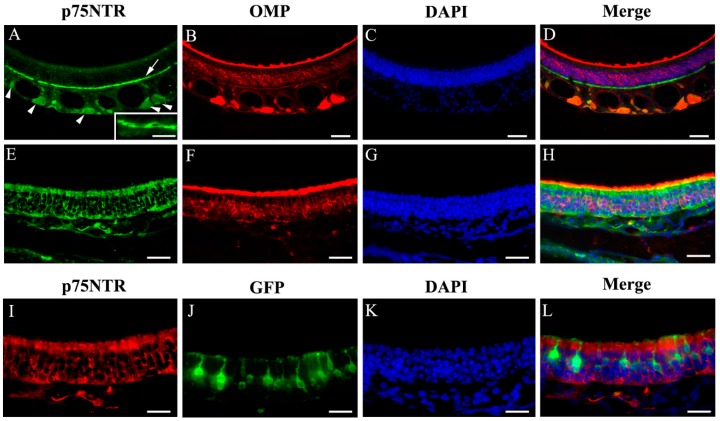
Immunoreactivity of p75NTR in different MOE regions. (**A**–**D**) Dorsal MOE. (**E**–**H**) Ventrolateral MOE. p75NTR immunolabel is present in the basal cell layer (**A**, indicated by arrow, inset shows an enlarged image of basal cells), in ensheathing cells in and around the olfactory nerve bundles (**A**, indicated by arrowheads) and in supporting cells in the ventrolateral region (**E**, green; **I**, red). OMP immunolabel is shown (**B**,**F**, red). GFP signal in TRPM5-expressing OSNs and TRPM5-MCs was intensified using an anti-GFP antibody (**J**). No apparent colocalization of the GFP and p75NTR signal was found. Sections were stained with DAPI to show nuclei (blue, **C**,**G**,**K**). Scale bar: (**A**–**D**) 50 μm; (**E**–**H)** 30 μm; (**I**–**L)** 20 μm.

Immunolabeling of p75NTR has been performed by several laboratories. While positive labeling was consistently reported in olfactory ensheathing cells, p75NTR immunoreaction was also found inconsistently in other cell types within the MOE and in the OSN axon bundles [18,21,22]. To further examine the expression of p75NTR in the MOE, we performed immunolabeling of coronal sections (Figure 8A–D,E–H are images of co-immunolabeling of p75NTR and OMP from the dorsal and ventrolateral MOE, respectively) using an antibody targeting the p75NTR intracellular domain, which also recognizes the full length protein [46]. As expected, we observed strong p75NTR immunoreactivity around the olfactory nerve bundles in the lamina propria, indicating that p75NTR is expressed by the olfactory ensheathing cells in the lamina propria which are associated with the bundles, but the signal was also visible inside the bundles (Figure 8A arrowheads). In sections co-immunolabeled with the anti-OMP antibody, p75NTR immunoreactivity was absent in mature OSNs (Figure 8B,C, DAPI labeling; Figure 8D: merge). Interestingly, we also found that anti-p75NTR strongly labeled the cell layer immediately above the basal lamina, which are presumably basal cells. The basal cell label was especially strong in the anterior MOE, and is similar to the labeling shown by Steuer et al. [22] (Figure 8A pointed out by an arrow, see inset also). Surprisingly, we found that the p75NTR immunoreactivity showed regional differences, with stronger labeling in lateral and ventral regions of the MOE (including the ectoturbinate). In these areas, the anti-p75NTR antibody intensely labeled supporting cells surrounding the OSNs in the OMP+ neuronal layer (Figure 8E–H, images of p75NTR, OMP, DAPI and overlay). However, we did not observe p75NTR immunoreactivity in TRPM5-MCs or GFP positive TRPM5-expressing neurons (Figure 8I–L, immunolabeling images of p75NTR and GFP, DAPI and merge). These results revealed diverse and regional p75NTR expression in the MOE. 

## 3. Discussion

We have investigated the influence of TRPM5-MCs and chemical exposure on expression of the olfactory signaling proteins and gene transcripts of NTs and NTRs in the MOE and OB. Our immunolabeling results showed that chemical exposure did not differentially alter the immunolabeling intensity of Gγ13, AC3, and CNGA2 in *Skn-1a^−/−^* and TRPM5-GFP mice. Our qPCR analysis revealed that the expression levels of individual NTs and NTRs in the MOE and OB varied substantially. Under the vehicle-exposure condition, both TRPM5-GFP and *Skn-1a^−/−^* mice showed similar expression levels for NTs and NTRs in the MOE and OB, and similar trends in the relative abundance of different NTs and NTRs within each tissue. After a two-week chemical exposure, qPCR analysis showed a significant increase in *p75NTR* expression in the MOE of *Skn-1a^−/−^* mice, in contrast to the significant decrease found in TRPM5-GFP mice. Additionally, *BDNF, NGF* and *NT-4* expression in the MOE of chemical-exposed *Skn-1a^−/−^* mice all trended higher. Our qPCR using RNA samples extracted separately from anterior and posterior MOE further showed some region-specific effects of the chemical exposure, which differed between TRPM5-GFP and *Skn-1a^−/−^* mice. Additionally, we also performed complementary RISH and immunolabeling for the MOE expression of NTs and NTRs, which indicated different expression patterns of NTs and NTRs and cellular origins of these molecules. Collectively, our results demonstrate that the gene-transcript levels of some NTs and NTRs were influenced by the chemical exposure and to some degree by the presence of TRPM5-MCs, shedding light on the internal and external factors regulating their expression and function in the MOE.

### 3.1. Expression of NTs and NTRs in the MOE and OB of the TRPM5-GFP and Skn-1a^−/−^ Mice Exposed to Vehicle (Water)

Both TRPM5-GFP and *Skn-1a^−/−^* mice in the vehicle (water)-exposed groups showed the same expression trend across all NTs and NTRs and comparable individual expression levels. These data demonstrate that under vehicle-exposure conditions, which is similar to conventional housing, TRPM5-MCs do not significantly influence the expression of NTs and NTRs in the MOE and OB. This result is consistent with our recently published data that vehicle-exposed *Skn-1a*^−/−^ and TRPM5-GFP mice showed similar odor-evoked responses and olfactory-guided behavior [12]. These data also indicate that TRPM5-MCs are not the major source for NTs and NTRs in the MOE, which is in agreement with previously published reports that immunoreactivity of NTs and NTRs is primarily found in either OSNs, supporting cells or basal cells [18,21].

Among the NTRs, there was a borderline significant difference (two-tailed independent *t*-test, *p* = 0.053, *n* = 6) in the level of *p75NTR* expression in the MOE with a higher level in vehicle-exposed TRPM5-GFP than in *Skn-1a*^−/−^ mice, implying that TRPM5-MCs might, to some degree, modulate *p75NTR* expression. Currently, information on NT and NTR signaling in the TRPM5-MCs is completely missing. One possible explanation is that *p75NTR* might be involved in the development and maintenance of cholinergic TRPM5-MCs since cholinergic neurons in the brain require NTs for proliferation, differentiation and maturation [47,48]. TRPM5-MCs differentiate from a distinct progenitor lineage expressing the transcription factor ASCL3 [15]. The lack of TRPM5-MCs in *Skn-1a*^−/−^ mice may diminish such a need for such NT-mediated regulation. Another possible explanation is that *p75NTR* expression level is regulated by activity of supporting cells which is modulated by acetylcholine as shown in our previous study [9,10]. p75NTR immunolabeling has been shown to present in supporting cells by Steuer et al. [22] and our current study.

### 3.2. Changes in the Expression Levels of NTs and NTRs in the MOE and OB of Chemical-Exposed TRPM5-GFP and Skn-1a^−/−^ Mice

For chemical-exposed TRPM5-GFP mice, our data obtained from the whole MOE sample showed significantly reduced *p75NTR* expression while the expression levels of all NTs and NTRs remained relatively unchanged, when compared to those of vehicle-exposed TRPM5-GFP mice (Figure 3A). Since upregulated *p75NTR* expression is closely associated with brain and olfactory system recovery from injury [22] and activation of Trk receptors by NTs promotes neuronal growth and survival [49,50], these results are concurrent with both our current and previous findings that there is no global morphological change in the MOE after the two-week chemical exposure [12]. The data also imply that upregulated activity of NTs and NTRs is not required for TRPM5-MCs-mediated MOE functional maintenance during chemical exposure.

What is the potential biological significance of reduced *p75NTR* expression in the MOE of chemical-exposed TRPM5-GFP mice? p75NTR forms a complex with individual NTRs as well as non-neurotrophin receptors sortilin and Nogo and interacts with numerous ligands to influence a wide range of neuronal activities such as neuronal survival, neurite outgrowth, and also apoptosis [49,51]. It is well known that p75NTR plays an important role in the development and function of the peripheral sensory neurons [52]. Because other NTs and NTRs are present in the MOE, down-regulation of *p75NTR* expression may reduce the formation of p75NTR-Trk complexes, consequently discouraging the survival of presumably worn-out or unhealthy OSNs due to the two-week chemical exposure, promoting cell turnover to maintain the MOE olfactory sensitivity of TRPM5-GFP mice. p75NTR can also form complex with sortilin [53], and when activated by proNGF, directly induces apoptosis. Currently, it is not known whether sortilin is expressed in the MOE.

Intriguingly, *p75NTR* was significantly upregulated in chemical-exposed *Skn-1a^−/−^* mice when TRPM5-MCs were absent in the MOE. This opposite change strongly indicates that TRPM5-MCs are an important factor regulating *p75NTR* expression in the MOE in response to chemical exposure. We also found a concomitant increase in expression of *BDNF*, *NGF*, *NT4* expression, although the differences between chemical-exposed and vehicle-exposed *Skn-1a^−/−^* mice did not reach statistical significance in RNA samples from the whole MOE. Similar trends were obtained from mRNA samples of the anterior MOE (not significant) and posterior MOE (significant for *BDNF* and *NT4*) of *Skn-1a^−/−^* mice after chemical exposure. Therefore, it is possible that the NT pathways in the MOE of chemical-exposed *Skn-1a^−/−^* mice are upregulated to compensate for the lack of TRPM5-MC-mediated protective modulation, which is important for MOE functional maintenance during a chemical challenge. Together, these results suggested possible crosstalk between TRPM5-MCs and neurotrophin pathways in the MOE.

We attempted to differentiate the regional impact of chemical exposure by performing qPCR using RNA samples extracted separately from either posterior or anterior MOE for qPCR analysis. The results showed consistent trends of expression levels across all the NTs and NTRs in both regions. Additionally, the results revealed a regional effect of the chemical exposure on some NTs and NTRs that differed between the two mouse lines. Further experiments are needed to verify this effect and to understand the biological meaning of the difference.

### 3.3. Discrepancy Between Different Approaches and Impact of Chemical Exposure Strength.

Our coronal MOE sections, which we obtained following our manual deboning method [54], allowed us to examine immunolabels in dorsal, ventral, medial and lateral regions of the MOE within a single section. We were surprised to observe strong regional difference in the p75NTR immunoreactivity in basal cells and supporting cells while labeling of ensheathing cells remained relatively consistent. In an earlier study, Feron et al. [21] indicated non-fixed tissue without antigen retrieval yielded a better p75NTR labeling. In trial experiments, we conducted two different types of antigen retrieval, which weakened the signal. Our deboning method also avoids chemical-based decalcification, which affects immunolabeling of some antibodies. Therefore, the discrepancy in p75NTR labeling could be a result of regional differences in addition to the previously identified factors [21].

Unlike the qPCR results obtained from the whole MOE samples, neither the anterior or posterior regions of chemical-exposed TRPM5-GFP showed a significant reduction in *p75NTR* expression. Factors contributing to this discrepancy are unknown, and it could potentially be due to the smaller sample size in the regional qPCR. Additionally, a significant reduction in BDNF and NT3 expression was found in the anterior MOE after the chemical exposure, but not in whole MOE samples. Since the anterior MOE is considerably smaller than the posterior MOE, it is possible that the differences were masked in the whole sample.

We also did not observe *BDNF* gene transcripts in the MOE in our RISH study although we tried two different riboprobes and positive labeling was found in OB sections. The causes of this inconsistency are currently not known but one contributing factor may be the relatively lower sensitivity of the RISH approach as compared to qPCR. Several previous studies have shown that *BDNF* is expressed in the MOE [21,33,34,55]. The potentially low level of *BDNF* expression found previously does not necessarily conflict with the strong expression of its high-affinity receptor (TrkB) in the MOE since NTs can undergo retrograde trafficking from the OB to the MOE, and BDNF is known to act on OSN axon terminals to promote their survival [42]. Additionally, TrkB can also be activated by NT-4 and our RISH analysis showed its expression throughout the MOE, which is consistent with previous reports [21].

Finally, although our results showed changes in NT and NTR expression levels after the two-week chemical exposure, the differences between TRPM5-GFP and *Skn-1a*^−/−^ mice are not drastic. These outcomes are expected as we chose relatively mild exposure conditions, aiming to investigate the molecular adaptive plasticity without inducing significant epithelial damage and subsequent the need for substantial repair. Severe chemical exposure would cause MOE damage and inflammation [56,57], which would most likely overpower the ability of TRPM5-MCs to maintain MOE function and engage distinct repair and regeneration mechanisms. It remains to be determined whether the changes in the NT and NTR expression found in our study after chemical exposure critically contribute to the TRPM5-MCs-mediated protective maintenance found in our previous study [12].

## 4. Materials and Methods

### 4.1. Animals

Two- to six-month-old male and female *Skn-1a*^−/−^ mice and TRPM5-GFP transgenic mice were used in this study. Both lines of mice are on C57BL/6 genetic background. The *Skn-1a*^−/−^ line of mice (RRID: IMSR_RBRC05254) was generated by Matsumoto et al. [58], and the complete loss of TRPM5-MCs in the MOE of *Skn-1a*^−/−^ mice was reported by Yamaguchi et al. [11]. We obtained breeder *Skn-1a*^−/−^ mice from Dr. Matsumoto’s laboratory (Monell Chemical Senses Center, Philadelphia, PA, USA) and maintained the colony through homozygous mating. Because there were no wild type littermates generated, we used TRPM5-GFP mice for control. The original breeding pair of TRPM5-GFP mice, in which the TRPM5 promoter drives GFP expression, enabling visualization of TRPM5-MCs [59], was kindly provided by Dr. Robert Margolskee. We have characterized TRPM5 expression in the MOE [7,60] and used TRPM5-GFP mice to study the TRPM5-MCs [9,10,12]. *Skn-1a*^−/−^ and TRPM5-GFP mice were group-housed in standard shoebox cages and randomly divided into either vehicle (water)-exposed or chemical-exposed groups. The age of the mice were matched for each cohort. All animal care and use procedures were conducted in accordance with the National Institutes of Health guide for the Care and Use of Laboratory Animals (2011) and approved by the Animal Care and Use Committee of the University, where the study was performed (approval number and date: WL01831518, June 25, 2015).

### 4.2. Chemical Exposure

Chemical exposure was performed as described in our previous study [12]. Briefly, age-matched *Skn-1a*^−/−^ and TRPM5-GFP mice were randomly divided into either vehicle-exposed or chemical-exposed groups. The expression levels of NT and NTR gene transcripts in their MOE and olfactory bulb (OB) were quantitatively determined using real-time qPCR. The odorant mixture used for the two-week exposure included ammonium hydroxide, ethyl acetate, propionic acid, and trimethylamine. The solutions for these chemicals were individually prepared in water, then mixed together to obtain a solution with final concentrations of 0.019, 0.075, 0.083, and 0.013 M, respectively. We selected the odorant concentrations for the exposure studies based on occupational health safety guidelines for irritant levels (www.cdc.gov/niosh/) and each RD_50_ value, which is the concentration that produces a 50% respiration rate decrease [37,61]. A small glass vial (either 1 cm diameter × 6.5 cm height or 1.8 cm diameter × 7 cm height) containing a piece of Kimwipe tissue paper with 4 mL of the mixture was placed in the cage. The odorant mixture was replaced daily, and the mice were exposed continuously for two weeks. Assuming the cage was closed, the gas-phase concentrations reached during daily exposures (derived by the vapor pressure of the mixture at 20–25 °C) were estimated to be 52 ppm for ammonium hydroxide, 180 ppm for ethyl acetate, 4.8 ppm for propionic acid, and 17 ppm for trimethylamine. In addition to the exposure to odorous volatiles, mice in the exposed group were also transferred to a new cage and exposed to chitin powder by agitating the powder around the cage with an air pump (250 mg/cage; Tokyo Chemical Industry Co., Ltd., Tokyo, Japan) for 10 min daily, during the two-week chemical-exposure period. Chitin is a characteristic component of fungal cell walls and exoskeletons of arthropods and insects, which exists abundantly in the natural environment [62]. During chitin exposure, an air pump was used to gently blow air around the cage to prevent the chitin powder from accumulating in the corner or being moistened by urine. Vehicle (water)-exposed *Skn-1a*^−/−^ and TRPM5-GFP mice were used as controls.

### 4.3. Immunocytochemistry

Tissue preparation. Vehicle- and chemical-exposed TRPM5-GFP and *Skn-1a^−/−^* mice were deeply anesthetized with tribromoethanol (Avertin 250 µg/g body weight), perfused transcardially with 0.1 M phosphate buffer (PB) followed by a PB buffered fixative containing 3% paraformaldehyde, 0.019 M L-lysine monohydrochloride and 0.23% sodium m-periodate [7]. The nose was harvested, post-fixed for 1.5 h, and transferred to 0.1 M phosphate buffer saline (PBS) with 25% sucrose overnight. The surrounding bones were then removed following our published method [54]. For stringent comparison, vehicle- and chemical-exposed noses were embedded in the same block in optimal cutting temperature (OCT) compound (Sakura Finetek USA Inc, Torrance, CA, USA). Fourteen-micron thick serial transverse sections were cut using a cryostat (Microm International, Walldorf, Germany), mounted onto plus-charged glass microscope slides (Globe Scientific Inc., Paramus, NJ, USA) and stored in −80 °C freezer until used.

Immunocytochemistry: Sections of the MOE were thawed at room temperature, rinsed 3 × 10 min in 0.1 M PBS and then incubated in PBS buffered blocking solution containing 2% normal donkey serum, 0.3% Triton X-100 and 1% bovine serum albumin for 1.5 h. Sections were then immunoreacted overnight or 48 h at 4 °C with primary antibodies against each of the following proteins: GFP (1:2000, Abcam cat# AB13970, Cambridge, UK), olfactory marker protein (OMP; 1:1000; #544-10001, Wako, Richmond, VA, USA), cyclic nucleotide gated channel A2 (CNGA2; 1:250; APC-045, Alomone Labs, Jerusalem, Israel), adenylyl cyclase III (AC3 or III; 1:200; SC-588, Santa Cruz Biotech, Santa Cruz, CA, USA), G-protein γ_13_ (Gγ_13_; 1:500; HPA046272, Sigma, St. Louis, MO, USA), and p75NTR (1:500, Millipore Cat.#ABN1655, Billerica, MA, USA). After incubation of the primary antibodies, sections were then washed and reacted with secondary antibodies (1:400, Alexa 488, Alexa 555, Alexa 647, Invitrogen, Carlsbad, CA, USA) for 1 h at room temperature. Sections were then stained with DAPI and mounted on slides with Fluoromount-G (SouthernBiotech, Birmingham, AL, USA). In controls for these experiments, primary antibodies were removed, resulting in negative labeling.

### 4.4. Real-Time Quantitative PCR

Primer design: Real-time qPCR primer sequences for each NT and NTR were obtained using Harvard PrimerBank [63], whereas primers used for RISH were designed using NCBI primer BLAST, as described previously [64]. Primers were synthesized by Invitrogen/Thermo Fisher Scientific (Carlsbad, CA, USA). The primer sequences, amplicon size, and NCBI GI numbers are shown in Table 1.

RNA extraction, cDNA synthesis, and agarose gel electrophoresis: These experiments were performed as described in our previous publications [28,40]. Briefly, animals were euthanized individually with CO_2_ asphyxiation followed by cervical dislocation and exsanguination by cutting open the heart. The main olfactory epithelium and the OB tissues were harvested and homogenized; total RNA was extracted using the NucleoSpin^®^ RNA Kit (Macherey-Nagel, Düeren, Germany). For experiments performed to investigate regional differences in gene expression, the olfactory tissue anterior to the olfactory turbinate was excised from the posterior olfactory tissue containing turbinate and posterior dorsal recess, and total RNA was separately extracted from both regions. cDNA synthesis was performed using the Maxima First Strand cDNA Synthesis Kit (Thermo Fisher Scientific, Waltham, MA, USA), according to manufacturer’s instructions. Briefly, 500 ng of total RNA was used as a template to prepare cDNA. A 1-μL aliquot of this cDNA was then used for each NT and NTR RT-PCR run. Amplicons were then run on a 2% agarose gel and the expected sizes were determined by comparing amplicons with GeneRuler DNA ladder (1 kb or/and 1 kb plus; Thermo Fisher Scientific). Amplicons were then extracted from the agarose gel using NucleoSpin^®^ Gel and PCR Clean-up Kit (Macherey-Nagel) before being ligated into the pGEMT-Easy vector (Promega, Fitchburg, WI, USA) to prepare the in situ hybridization riboprobes.

Real-time qPCR: To prepare reaction mixtures, 1 μL cDNA was added to 10 μL iTaq^TM^ Universal SYBR^®^ Green Supermix (Bio-Rad, Hercules, CA, USA) along with the forward and reverse primers for each gene; the total reaction volume was adjusted to 20 μL by adding RNase-free water. The reaction mixtures were loaded in clear Multiplate^®^ 96-Well PCR Plates (Bio-Rad) and sealed. The qPCR was performed using a Touch Thermal cycler C1000 with the CFX96 Real-Time System (Bio-Rad). The qPCR data were analyzed using the relative quantification method (2^−ΔΔ*C*t^), as described by Livak and Schmittgen [65]; mRNA expression of Gapdh was detected as an internal reference.

### 4.5. RISH Experiments

Tissue preparation, riboprobe synthesis, hybridization, detection, and image acquisition were performed as previously described [40]. Briefly, all solutions for RISH were prepared using 0.1% diethyl pyrocarbonate water (Sigma-Aldrich Co., St. Louis, MO, USA) and RNase-free conditions were maintained during the experiments using RNaseZAP^TM^ (Sigma-Aldrich Co.). As mentioned above, RNA riboprobes were synthesized by cloning RT-PCR amplicons into pGEMT-Easy vector, which was linearized and used as template to generate digoxigenin-UTP (DIG) sense and antisense probes. The primers used to generate the probes are described in Table 1. Additionally, two sets of primers were used to prepare two different BDNF RISH probes, including the primers that were used for BDNF qPCR (Table 1). One set of primers was adapted from Wüllner et al. [66]. For tissue preparation, mice were deeply anesthetized using tribromoethanol (Avertin, 250 µg/g body weight) and perfusion-fixed with a phosphate-buffered 4% paraformaldehyde (PFA). The noses were excised and post-fixed overnight with 25% sucrose for cryoprotection. The noses were then deboned, following our previously published protocol [54], and the MOE tissue was sectioned (14-µm thickness) using a cryostat and mounted onto charged microscope slides (Globe Scientific, Paramus, NJ, USA). For RISH, the mounted MOE sections were fixed in 4% PFA for 15 min, washed in phosphate-buffered saline (PBS), incubated in proteinase K solution (10 μg/mL in TE) for 7 min, fixed again in 4% PFA for 10 min, and acetylated for 10 min, followed by ethanol treatment for dehydration. Then, the riboprobes were dissolved in a hybridization solution (50% deionized formamide, 10 mM Tris-Cl [pH 8.0], 10% dextran sulfate, 1× Denhardt’s solution, 200 mg/mL tRNA, 0.6 M NaCl, 0.25% SDS, 1 mM EDTA) and applied to each slide (0.2 μg/mL of stock probe; 200 μL/slide). Slides were then placed in a moist hybridization chamber and incubated overnight in an incubation chamber preheated to 65 °C. Anti-digoxigenin-AP, Fab fragments of polyclonal antibodies from sheep (Roche, Basel, Switzerland, catalog number 11093274910; 1:1000 dilution) with 250 μL nitro blue tetrazolium/5-bromo-4-chloro-3′-indolyl phosphate (NBT/BCIP; 1 tablet/10 mL water; Sigma-Aldrich Co.) were used for probe detection. The antibody and NBT/BCIP mixture was applied to each slide and incubated in the dark until the control antisense riboprobe slides developed a purple color in the appropriate regions of the tissue sections. The slides with the sense riboprobe were monitored to avoid overexposure. Slides were then washed in PBS and mounted with Fluoromount-G (Southern Biotech, Birmingham, AL, USA) and covered with a coverslip.

### 4.6. Image Acquisition

Fluorescence images of immunolabeled sections were taken using an Olympus BX 61 epi-fluorescence microscope equipped with a spinning disc confocal unit using Slidebook 4.0 software (3I Corporation, Denver, CO, USA). Images of RISH signals in the MOE sections were acquired using the 10× and 40× objective lenses of an Olympus BX 41 compound microscope equipped with a Retiga 4000 R digital camera (QImaging, British Columbia, Canada). Q Capture Pro 7^TM^ software (QImaging) was used to capture the images

### 4.7. Statistical Analysis

A two-tailed independent *t*-test was used to compare the qPCR expression data for the mRNA levels of NTs and NTRs between control and *Skn-1a*^−/−^ mice. One-way ANOVA followed by Tukey’s post-hoc analysis was performed to determine statistically significant differences in the expression of NT and NTR gene transcripts in the MOE and OB of control and *Skn-1a*^−/−^ mice. A two-tailed independent *t*-test was also used to compare the gene-transcript levels after a two-week chemical exposure. In addition, two-way ANOVA was performed to compare the effects of a two-week chemical exposure on the level of NTs and NTRs in the MOE and OB of control and *Skn-1a*^−/−^ mice. All statistical analyses were performed using IBM SPSS software for Windows, Version 24.0 (Released 2016; IBM Corp., Armonk, NY, USA). For all tests, *p* < 0.05 was considered statistically significant. The bar graphs represent the means ± SD.

## 5. Conclusions

We have investigated the impact of chemical exposure on the expression of olfactory signaling protein and gene transcripts of NTS and NTRs known to play important role in olfactory maintenance. We did not see obvious difference in the intensity of AC3, CNGA2, and Gγ13 immunolabeling between chemical-exposed TRPM5-GFP and *Skn-1a^−/−^* mice. Thus direct reduction of olfactory signaling proteins is unlikely to be responsible for the functional deficits in chemical-exposed *Skn-1a^−/−^* mice found in our previous study [12]. We have also quantitatively determined the expression levels of NTs and NTRs in the MOE and OB in TRPM5-GFP and *Skn-1a*^−/−^ mice and impact of the two-week chemical exposure. Our results confirmed the coexistence of multiple NTs and NTRs in the olfactory system and provided new evidence that chemical exposure and the lack of TRPM5-MCs altered the expression levels of particular NTs and NTRs, without perturbing their overall expression trends. These relatively moderate results are likely due to the facts that OSNs and SCs, but not TRPM5-MCs, make up the major cell types in the MOE expressing NTs and NTRs, and that our chemical exposure conditions did not produce significant MOE damage [12]. To the best of our knowledge, this is the first investigation of the relationship between TRPM5-MCs and NTs and NTRs. Our results indicate potential crosstalk between two mechanisms that play a role in maintaining olfactory function, especially in a challenging chemical environment.

## Figures and Tables

**Table 1:** Oligonucleotide primer sequences used for real-time qPCR and RISH analyses of gene transcript expression of NTs and NTRs.

Gene Name	NCBI GI Number	Primer Sequence (5’ = Forward; 3’ = Reverse)	Application and Primer Bank ID	Amplicon Size (bp)
*TrkA (NTRK1)*	568922048	5’: CAGTCTGATGACTTCGTTGATGC 3’: CTCTTCACGATGGTTAGGCTTC 5’: AAGGCGGGCGCCGCCGCGAT 3’: TCTCAACTCCCCCAGGCCCT	qPCR 169234625c1	243
			Probe for RISH	300
*TrkB (NTRK2)*	68215969	5’: CTGGGGCTTATGCCTGCTG 3’: AGGCTCAGTACACCAAATCCTA 5’: GACAGGCTCAGCCTCTGGTA 3’: TTGAGCCACATGATGTCGCA	qPCR 6679150a1	100
			Probe for RISH	630
*TrkC (NTRK3)*	755520797	5’: CTGAGTGCTACAATCTAAGCCC 3’: CACACCCCATAGAACTTGACAAT 5’: GAATAGTCTCATGGCATATC 3’: CATCCAATGCAGACACTAGA	qPCR 33413412a1	157
			Probe for RISH	210
*p75NTR (TNFR)*	70794802	5’: CTAGGGGTGTCCTTTGGAGGT 3’: CAGGGTTCACACACGGTCT 5’: TGCAATTAGTAGAAGGACCCCACC 3’: TACACAGGATGCAAAGGGGA	qPCR 15082265a1	140
			Probe for RISH	264
*NGF*	162951830	5’: TGATCGGCGTACAGGCAGA 3’: GCTGAAGTTTAGTCCAGTGGG 5’: AAACTTCAGCATTCCCTTGA 3’: CCTGTTGAAAGGGATTGTAC	qPCR 162951830c1	87
			Probe for RISH	231
*BDNF*	34328441	5’: TCATACTTCGGTTGCATGAAGG 3’: AGACCTCTCGAACCTGCCC 5’: GAAAGTCCCGGTATCCAAAG 3’: CCAGCCAATTCTCTTTTT	qPCR and RISH 34328442a1	137
			Probe for RISH	181
*NT-3*	568941025	5’: GGAGTTTGCCGGAAGACTCTC 3’: GGGTGCTCTGGTAATTTTCCTTA 5’: TACAGGTGAACAAGGTGATG 3’: CCTGCTCTGGTTCCCTGGGT	qPCR 6679144a1	117
			Probe for RISH	240
*NT-4*	755521409	5’: TGAGCTGGCAGTATGCGAC 3’: CAGCGCGTCTCGAAGAAGT 5’: CTCTTCCTGCTGGAGGCCGG 3’: GCTTTCGGCCTTGCAGCGCGT	qPCR 30353913a1	147
			Probe for RISH	261

## Abbreviations

Ach	Acetylcholine
ANOVA	Analysis of variance
BCIP	5-bromo-4-chloro-3′-indolyl phosphate
BDNF	Brain-derived neurotrophic factor
ERK	Extracellular signal-regulated protein kinase
GFP	Green fluorescent protein
HBC	Horizontal basal cell
MC	Microvillous cell
MOE	Main olfactory epithelium
NBT	Nitro blue tetrazolium
NGF	Nerve growth factor
NT	Neurotrophin
NT-3	Neurotrophin-3
NT-4	Neurotrophin-4
NTR	NT receptor
OB	Olfactory bulb
OSN	Olfactory sensory neuron
PBS	Phosphate-buffered saline
PFA	Paraformaldehyde
PI3K	Phosphatidylinositol-4,5-bisphosphate 3-kinase
PLCγ	Phospholipase Cγ
qPCR	Quantitative polymerase chain reaction
SC	Supporting cell
SD	Standard deviation
RISH	RNA in situ hybridization
RT-PCR	Reverse transcription polymerase chain reaction
Trk	Tropomyosin receptor kinase
TRPC6	Transient receptor potential channel C6
TRPM5-MC	Transient receptor potential channel M5-expressing microvillous cell
